# Course, outcome and complications in a single centre cohort of 53 indian children with systemic onset juvenile idiopathic arthritis with a minimum follow up of 3 years

**DOI:** 10.1186/1546-0096-12-S1-P65

**Published:** 2014-09-17

**Authors:** Mansi Dewoolkar, Rolando Cimaz, Raju P Khubchandani

**Affiliations:** 1 Pediatrics, Jaslok Hospital & Research Center, Mumbai, India; 2 Pediatrics, AOU Meyer, Florence, Italy

## Introduction

Systemic-onset Juvenile Idiopathic Arthritis (SOJIA) is not rare in India, where biotherapies are unaffordable.Data on its course, outcome and complications are scarce.

## Objectives

We describe this data in a monocentric cohort of 53 patients, followed up for at least 3 years.

## Methods

A pre-biologic era Italian study where one of the authors (RC) had participated, formed a template and comparator. After ethics committee approvals and consents, a cohort of 53 consecutive patients diagnosed with SOJIA before 10-2009 using the ILAR criteria were followed up until 09-2012. At each visit, general (including growth parameters) and articular examination, laboratory parameters (CBC, ESR , liver enymes) and ongoing treatment were entered in a customized database.Course was classified as monocyclic (single episode) polycyclic (multiple episodes with remissions in between) and persistent (continuous articular/systemic disease activity). At last visit, outcome was studied with respect to remission (Wallace criteria) and Steinbrocker functional classification. Juvenile Arthritis Damage Index (JADI) measured on 20/53 patients.

## Results

In the 53 patients studied (35-M,18-F), 21 constituted an inceptional cohort and 32 were referred to our center with prior diagnosis / treatment. Mean age at diagnosis was 6.3 years (range 4m-14y), mean follow up period was 5.5 years (range 3-10 years) and mean time from onset of symptoms to diagnosis was 8.5 months (range 2 wks-7 years). Forty four patients received NSAIDs, 52 oral corticosteroids and 34 required pulses of methylprednisolone with intra-articular triamcinolone acetonide being used in 14. Methotrexate was used in 50 patients, other DMARDs in 25(hydroxychloroquine, leflunomide, cyclosporine, cyclophosphamide, thalidomide) and 5 received biologics briefly (etanercept-2, tocilizumab-3). Nine had a monocyclic, 31 intermittent and 13 persistent course. At last visit, 9/9 patients of the monocyclic group,17/31 in the intermittent group and 3/13 in the persistent group were in remission. Patients diagnosed within 6 months from disease onset were more likely to have a monocyclic / intermittent than a persistent course, compared to those diagnosed later. 33/53 suffered from complications of the disease and /or drug. MAS was observed in 5 and death occurred in 1, due to hepatic encephalopathy complicating viral hepatitis A.Three required orthopedic surgeries for residual deformities. All children in the monocyclic group belonged to Steinbrocker class 1 at last visit. Of 31 in the intermittent group, 27 belonged to class 1 and 4 to class 2. In 13 of the persistent group, 7 belonged to class 1, 4 to class 2 and 2 to class 3. JADI was performed on 20/53 patients.9 had significant articular damage. The range of JADI-Articular was 0-25/72 (median-6) and the range of JADI- Extra-articular was 0-4/17 (median-1).

## Conclusion

Drug regimens comprising NSAIDs, steroids (oral/intra-articular),methotrexate and other DMARDs still form the mainstay of SOJIA treatment in India in contrast with the developed world where biologics have come to the forefront. As a result 33/53(65%) patients showed drug and/or disease-related complications. Delay in diagnosis is a major problem and is associated with a persistent course. Children with monocyclic and intermittent course have the best functional outcome. JADI is an easily applicable bedside tool to evaluate both articular and extra articular damage of SOJIA.

## Disclosure of interest

None declared.

